# *Moringa oleifera* Seeds and Oil: Characteristics and Uses for Human Health

**DOI:** 10.3390/ijms17122141

**Published:** 2016-12-20

**Authors:** Alessandro Leone, Alberto Spada, Alberto Battezzati, Alberto Schiraldi, Junior Aristil, Simona Bertoli

**Affiliations:** 1 International Center for the Assessment of Nutritional Status (ICANS), University of Milan, Via Sandro Botticelli 21, 20133 Milan, Italy; alberto.battezzati@unimi.it (A.B.); simona.bertoli@unimi.it (S.B.); 2 Department of Food, Environmental and Nutritional Sciences (DeFENS), University of Milan, Via Mangiagalli 25, 20133 Milan, Italy; alberto.schiraldi@unimi.it; 3 Department of Agricultural and Environmental Sciences-Production, Landscape, Agroenergy (DISAA), University of Milan, Via Celoria 2, 20133 Milan, Italy; alberto.spada@unimi.it (A.S.); junior.aristil@unimi.it (J.A.)

**Keywords:** *Moringa oleifera*, seeds, oil, phytochemical compounds, pharmacology, human health

## Abstract

*Moringa oleifera* seeds are a promising resource for food and non-food applications, due to their content of monounsaturated fatty acids with a high monounsaturated/saturated fatty acids (MUFA/SFA) ratio, sterols and tocopherols, as well as proteins rich in sulfated amino acids. The rapid growth of *Moringa* trees in subtropical and tropical areas, even under conditions of prolonged drought, makes this plant a reliable resource to enhance the nutritional status of local populations and, if rationalized cultivation practices are exploited, their economy, given that a biodiesel fuel could be produced from a source not in competition with human food crops. Despite the relatively diffuse use of *Moringa* seeds and their oil in traditional medicine, no pharmacological activity study has been conducted on humans. Some encouraging evidence, however, justifies new efforts to obtain clear and definitive information on the benefits to human health arising from seed consumption. A critical review of literature data concerning the composition of *Moringa* oil has set in motion a plan for future investigations. Such investigations, using the seeds and oil, will focus on cultivation conditions to improve plant production, and will study the health effects on human consumers of *Moringa* seeds and their oil.

## 1. Introduction

*Moringa oleifera* (Moringaceae) is a fast-growing softwood tree indigenous to sub-Himalayan tracts of Northern India. It is one of 13 species within the same genus, and has become the most diffuse in tropical and subtropical areas at altitudes up to 2000 m [1]. Nowadays, *M. oleifera* is mainly found in the Middle East and in African and Asian countries, but, due to its adaptability, it is spreading to other areas, especially tropical and subtropical lands affected by drought.

All parts of the *Moringa* tree (leaves, seeds, roots and flowers) are suitable for human and animal consumption. The leaves, which are rich in protein, minerals, β-carotene and antioxidant compounds, are used not only for human and animal nutrition but also in traditional medicine [1,2]. The seeds, instead, have attracted scientific interest as *M. oleifera* seed kernels contain a significant amount of oil (up to 40%) with a high-quality fatty acid composition (oleic acid > 70%) and, after refining, a notable resistance to oxidative degradation [3]. The oil is known commercially as “Ben oil” or “Behen oil”. Its properties make it suitable for both human consumption and commercial purposes. Indeed, *Moringa* oil could be a good substitute for olive oil in the diet as well as for non-food applications, like biodiesel, cosmetics, and a lubricant for fine machinery. Moreover, after oil extraction, the seed cake can be used in waste water treatment as a natural coagulant [4] or as an organic fertilizer to improve agricultural productivity [5].

In this article, we conducted a critical review of literature data concerning the composition of *Moringa* seeds and oil. Moreover, we reviewed all studies reporting pharmacological activity arising from *Moringa* seed consumption, as well as other activities that can have an impact on human health. We have highlighted gaps in current knowledge, as such gaps represent a starting point for planning future investigations. To the best of our knowledge, this is the first critical review focused on the chemical and nutritional composition of *M. oleifera* seeds and oil, their phytochemical content and their potential uses for human health. Previous reviews discussed the characteristics and properties of *M. oleifera* without clearly attributing them to specific plant parts. However, the individual treatment of each part is fundamental because of its different composition and properties. For this reason, the present review includes only those articles that report studies on *M. oleifera* seeds and their oil.

## 2. Cultivation for Seed Production

There are two main ways of obtaining *M. oleifera* plants: sowing and the use of cuttings [1].

For seed production, sowing is preferred as improved varieties can be selected for cultivation, ensuring proper and profitable production [6]. Seed production, according to harvest and management practices [1], requires a low density plantation (typically, 2.5 m × 2.5 m, or 3 m × 3 m) with a triangular pattern [7], although 1.2 m along a row and 5 m between rows also seems suitable for satisfactory yields [8]. For leaf production, the spatial distribution in planting can vary: intensive (spacing from 10 cm × 10 cm to 20 cm × 20 cm), semi-intensive (spacing 50 cm × 100 cm), or integrated into an agroforestry system (spacing distance of 2–4 m between rows).

Normally, *Moringa* seeds are sown during the rainy season and can germinate and grow without irrigation, but for commercial purposes, irrigation through a drip system is recommended, allowing seed production during the dry season as well. Should irrigation be employed, its conditions depend on the cultivation area. In a recent study, Muhl et al. [9], who used a rainfall exclusion method, administered three irrigation treatments: 900, 600 and 300 mm per annum through drip irrigation, simulating three total annual rainfall amounts. The study showed that a restricted water supply caused no stress to the trees during the reproductive stages; moderate water prior to floral initiation could be beneficial, stimulating flower initiation, while ample irrigation thereafter ensured better fruit set and greater yield.

Although *M. oleifera* can produce a large quantity of seeds when fertilization is adequate, there has been no exhaustive research on this issue. Several studies [7,10,11], focused on leaves or biomass production, suggest that the vegetative growth of *Moringa* is best supported by 120 kg N:P:K/ha. Fertilization must be done during soil preparation before sowing, and when the trees are at the onset of the growth period, i.e., just before the rainy season. Manure or compost can be used instead of chemical fertilizer.

## 3. Flower Biology and Reproduction

*Moringa* inflorescences are loose panicles produced in axillary, drooping 10 to 25 cm in length. The bisexual zygomorphic flowers are up to 12 mm long and are white or cream in colour, fragrant, and have 5 pale green sepals, 5 white petals, 5 stamens with anthers and 5 without (staminoid) [12]. The flowers are highly cross-pollinated due to delayed stigma receptivity, and successful pollination requires a large number of insects [13].

The fruit is a trilobite capsule (referred to as a pod), 20–60 cm in length, and it ripens about three months after flowering. The pods become brown and dry at maturity and split open into 3 parts longitudinally. Each pod usually contains 12 to 35 round seeds, 1 cm in diameter. The seeds have a brownish semi-permeable hull with three papery whitish “wings” set around it at 120-degree intervals. A single tree can produce from 15,000 to 25,000 seeds, each weighing, on average, ca. 0.3 g [14]. Early flowering varieties produce pods in six months, while other varieties require more than one year.

## 4. Production

Seed production [15] varies tremendously. Ndubuaku et al. [16] studied *M. oleifera* yields across Nigeria and reported 4 to 24 tons of seeds/hectare, depending on location, soil type, vegetation and climate conditions. There is little information about other countries’ production.

The present-day interest in *M. oleifera* is not its commercial value, it is its multipurpose uses and reliability in guaranteeing good yield. Traditionally, its cultivation is almost exclusively for fresh pod production where there is low population density [17]. This reliability is not the case for other crops in countries where people are often faced with famine due to crop losses, and where whole populations are very likely to suffer nutritional deficiencies. However, before proposing *Moringa* cultivation for such countries, studies are needed as little information is available on *Moringa* seed yield under a high-density orchard design.

Recently, Ayerza [18] performed a comparative trial in the four ecosystems of Argentina and Bolivia (Arid Chaco, The Yungas Tropical Forest, Sub-Humid Chaco Lowland, Tropical Forest) to determine the seed yield and oil of Periyakulam-1 (PKM-1), an early *Moringa* variety provided by the Horticultural Research Station of the Tamil Nadu Agricultural University (TNAU) in India. Differences between years and localities were investigated. Ayerza showed that over the three-year field trials, the oil percentages for seeds produced at Arid Chaco were significantly higher than at Sub-Humid Chaco; however, the seed/tree yields and the oil/tree content among the ecosystems did not differ significantly. Interestingly, when oil percentage per tree and seed yield/tree were combined, the trees from Sub-Humid Chaco and Yungas Tropical Forest yielded a significantly higher oil content than from the Arid Chaco trees, suggesting that location affects yield, presumably due to one or more environmental factors such as temperature, light, soil type and available nutrients.

In a previous study, Ayerza [8] performed an experimental trial in a semi-commercial *Moringa* plantation in a subtropical north-western region of Argentina. Ayerza compared two *Moringa* varieties: PKM-1 and an African accession from Tanzania of unknown selection pressure. Pods per tree, seeds per pod, weight of seed per pod, kernel weight, kernel oil content and fatty acid composition were determined. He found that seeds per tree, oil yield per tree, and seed weight were higher for the PKM-1 cultivar trees than for the African cultivar, suggesting that the genotype from India would be more economically useful in a subtropical environment. He also found that the oil from the two cultivars had a practically identical fatty acid composition, the percentages ranging from 31.8% to 40.8% depending on the different years and trees.

The marked difference between the component yields of PKM-1 and the Africa cultivar indicates that the highest levels of genetic diversity can be expected within PKM-1 populations, which is in line with previous studies [19]. Such findings support PK1 as a good candidate for the development of improved *Moringa* varieties.

## 5. Chemical Characteristics of Seeds and Oil

**Figure 1:**
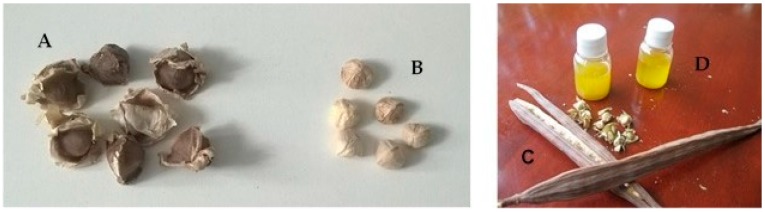
Seeds (**A**), kernels (**B**), fruits (**C**) and oil (**D**) of *Moringa oleifera*.

*M. oleifera* seeds are globular, about 1 cm in diameter. They are three-angled, with an average weight of about 0.3 g, 3-winged with wings produced at the base of the seed to the apex 2–2.5 cm long, 0.4–0.7 cm wide; the kernel is responsible for 70%–75% of the weight [20] (Figure 1).

Oil is the main component of the seed and represents 36.7% of the seed weight. The oil can be extracted almost entirely by solvent extraction, generally n-hexane, whereas less yield is obtained by cold press extraction. In fact, only 69% (on average) of the total oil contained in seeds can be extracted by cold press [21,22,23]. Among rural dwellers, the edible oil is extracted by boiling de-husked seeds with water, and collecting the oil from the surface of the water [22]. Apart from the oil, the seed has a high protein content, on average 31.4%, whereas carbohydrate, fibre and ash contents are 18.4%, 7.3% and 6.2%, respectively. Thus, the defatted seeds of *M. oleifera* could provide an economical source of protein for use as a food supplement to traditional diets to increase protein intake. Furthermore, like the protein fraction, *M. oleifera* seeds have a high content of methionine and cysteine, close to that reported for milk and eggs [24]. Therefore, they can be consumed together with legumes which are deficient in sulphur amino acids. Moreover, *M. oleifera* seeds seem to be free of trypsin inhibitor and urease activity, confirming the high protein digestibility (93%) of *M. oleifera* seeds [24,25]. Table 1 shows the chemical composition of the *M. oleifera* seed.

Table 2 summarizes the physical and chemical characteristics of *M. oleifera* oil. It is liquid at room temperature and golden yellow in colour. The extraction method does not in any way affect the density and refractive index of the oil, and both are similar to those of olive oil [30]. Instead, the smoke point is approximately 11 °C higher than that of olive oil [21,31], suggesting a greater stability during the frying process. The oil obtained by cold pressure extraction is higher in viscosity and acidity than that obtained by solvent-extraction. This higher viscosity is due to the water bound in the oil during extraction [31], while the higher acidity is attributed to the water added during the milling of the seeds prior to cold pressing. Indeed, the water addition enhances the lipolytic enzyme action [32] and prolongs the contact of the seed (milled before cold pressing) with air and temperature. Nevertheless, the acidity of the cold-pressed oil is generally moderate, indicative of its good resistance to hydrolysis. The iodine number is lower than that of olive oil as *Moringa* oil is less unsaturated than olive oil [30]. Finally, the saponification value, regardless of the extraction method, is similar to that of olive oil [30].

Table 3 shows the fatty acid composition of *M. oleifera* oil. The saturated fatty acid content is 21.18%, with palmitic acid dominating, closely followed by behenic, stearic and arachidic acids. The high behenic acid content is the reason why the oil is known commercially as “Ben” or “Behen” oil. Small traces of cerotic, lignoceric, myristic, margaric and caprylic acids are also reported in *M. oleifera* seed oil. The oil contains a high level of monounsaturated fatty acids, up to an average of 76.73%. Oleic acid is the predominant fatty acid, and accounts for 73.57% of the total fatty acids. Further monounsaturated fatty acids present in the oil are gadoleic and palmitoleic acids. Small traces of erucic acid are reported by some studies. There is a very low content of polyunsaturated fatty acids, on average 1.18%, and the content of linoleic and linolenic acids is 0.76% and 0.46%, respectively. In addition, the oil’s fatty acid composition does not seem to be particularly affected by the extraction method. Only one study reported a small increment of the stearic and myristic acid content in solvent-extracted oil compared to oil obtained by cold pressure [23]. On the other hand, the agro-climatic characteristics of the cultivation area and the *M. oleifera* variety cultivated could be the reason for some differences in the fatty acid composition of the oil. Nevertheless, the present fatty acid composition shows that *M. oleifera* seed oil falls in the category of high-oleic oils, and contains a high monounsaturated to saturated fatty acids ratio (MUFA/SFA). The MUFA/SFA ratio is characteristic of several oils, particularly olive oil, and has been associated with a reduced risk of all-cause mortality, cardiovascular mortality, cardiovascular events, and stroke [33]. Therefore, *M. oleifera* seed oil could be an acceptable substitute for olive oil as the main dietary fat in countries where the tree grows. *M. oleifera* seed oil has a monounsaturated fatty acid content similar to that of olive oil [30,34], but from a nutritional point of view, a lower content of polyunsaturated fatty acids is a limiting factor, which needs to be offset by the consumption of alternative sources rich in polyunsaturated fatty acids. However, from a technological point of view, the low content of polyunsaturated fatty acids ensures greater resistance and stability to oxygen.

With regard to the sterol composition, *M. oleifera* seed oil differs in composition from olive oil and other conventional vegetable oils used in cooking [30].

Table 4 shows the sterol fractions present in *M. oleifera* seed oil.

The sterol fractions of the oil consist mainly of β-sitosterol, stigmasterol, campesterol and Δ^5^-avenasterol, these accounting for 92% of the total sterols. Other sterol fractions are only present in trace amounts. The composition of the sterols is not affected by the extraction method; however, the degumming process involves a reduction in the sterol compounds. Other factors, such as plant variety and agro-climatic conditions of cultivation, could affect the sterol composition of the oil [26,28]. The sterol fraction is of interest for its possible involvement in the metabolism of cholesterol, lowering the circulating level of LDL cholesterol [36,37,38]. Other studies also suggest an antidiabetic potential of β-sitosterol [39]. However, dietary results concerning the impact of plant sterols on cardiovascular risk are still conflicting and inconclusive [40].

*M. oleifera* seed oil is characterized by a high tocopherol content, consisting of α-, γ- and δ-tocopherols. Table 5 shows its tocopherol profile.

The tocopherol content could depend on the extraction method used to obtain the oil; however, results in this context are controversial. In fact, although one study showed the content of α- and δ-tocopherol to be significantly higher in oil obtained by cold pressing [21], another study, by the same authors, found a marked similarity in the α- and δ-tocopherol content of oils obtained by cold pressing and solvent extraction [31]. Nevertheless, the tocopherol content is higher than that of other oils. The average content of α-tocopherol, which has the greatest vitamin E potency, reaches 132.3 mg/kg, whereas the average content of the γ- and δ-tocopherols is 63.9 mg/kg and 81.2 mg/kg, respectively, also higher than contents reported for oils from seeds of other *Moringa* family species [41,42]. Such a high tocopherol content can be expected to contribute good oxidative stability and protection to *M. oleifera* oil during its storage and processing [29].

Besides its use in human nutrition, *M. oleifera* oil also suits non-food purposes. Indeed, *M. oleifera* oil has attracted the attention of researchers because of its potential in the production of biodiesel. A recent study [43] conducted in Australia reports that approximately 3030 kg of oil can produce 1000 L of biodiesel. Note that an equivalent of 3.03 tonnes/ha of oil seeds can be harvested from dry land, and 6.06 tonnes/ha from irrigated land. Moreover biodiesel production with *Moringa* seed oil is not in direct competition with existing farmlands or food crops (second generation), and as *Moringa* can be grown on degraded land, studies suggest that *Moringa* biodiesel is an acceptable substitute for fossil fuels, even compared to biodiesel derived from vegetable oils of other species [35,44].

## 6. Phytochemistry and Medicinal Uses

In addition to their macronutrient composition (Table 1), making them attractive for oil production and as an economic source of protein, the seeds of *M. oleifera* have been extensively studied for their content in secondary metabolites, also making them interesting for medical purposes. Several studies have found good antioxidant activity and have isolated phytochemical compounds that, because of their biological properties, can be used as nutraceutical molecules [45,46]. The total phenolic content of *M. oleifera* seeds has been found to be in the range of 4581–4953 mg/100 g [45,47], similar to leaf amounts [2]. The flavonoids are represented by catechin, epicatechin, quercetin and kaempferol [45,47], present mainly in the bound form [47]. Moreover, several phenolic acids have been identified, gallic acid predominating, followed by ellagic and caffeic acids. Phenolic acids, like *p*-coumaric, vanillic, protocatechuic, ferulic and cinnamic acids, have also been identified in *M. oleifera* seeds, but in smaller amounts [45,47]. Interestingly, *M. oleifera* seed also contains important bioactive compounds including alkaloids, glucosinolates, isothiocyanates and thiocarbamates.

Table 6 shows all the phytochemicals isolated from the seeds of *M. oleifera*.

All these compounds could be responsible for the pharmacological properties attributed to *M. oleifera* seeds.

Folk medicine uses raw or crushed *M. oleifera* seeds as a decoction for treating stomach pain, ulcers, poor vision, joint pain and for aiding digestion [57]. The seed extract has been found to possess good antimicrobial activity against numerous bacterial and fungal species [47,52,58,59,60,61]. Many of the phytochemical compounds isolated from the seeds are able to inhibit the growth of certain pathogenic microorganisms responsible for human infections [52,58,59]. For this reason, some authors have speculated the use of these phytochemicals as an alternative to traditional therapies as they can be pharmacologically effective with low or no side effects [61,62]. The seeds’ antimicrobial activity is also related to the presence of a short cationic protein. This protein, known as the *M. oleifera* cationic protein, causes bacterial cell damage through rapid flocculation and the fusion of cell inner and outer membranes. Because of their antimicrobial activity, *M. oleifera* seeds are used as nature-based solutions for the problem of water purification in developing countries, using them as an alternative to Western methods. Studies have shown that this simple filtering method reduces not only water pollution but also harmful bacteria.

The seeds of *M. oleifera* have been found to be good antioxidants, able to reduce oxidative damage associated with aging and cancer [45]. Many of the bioactive compounds isolated from *M. oleifera* seeds have been found to be potential antitumor promoters [46,53]. However, recent study results found that the ethanol extract of *M. oleifera* seeds had no significant effect in inhibiting the proliferation of breast and colorectal tumour cells [63]. Nevertheless, a recent study observed a cytotoxic effect of *M. oleifera* oil in several cancer cell lines [64].

One study reported that *M. oleifera* seeds possess hepatoprotective, anti-inflammatory and anti-fibrotic properties against CCl_4_-induced liver damage and fibrosis [65]. After intoxication with CCl_4_, rats simultaneously receiving treatment with *Moringa* seed ethanol extract (1 g/kg body weight) for 8 weeks, compared with untreated rats, showed: (i) lower serum levels of AST and ALT and higher serum albumin levels, indicating better liver synthesis function; (ii) lower globulin levels, reduced myeloperoxidase activity as well as lower hepatic infiltration of inflammatory cells, indicating less inflammation; and (iii) lower hepatic levels of hydroxyproline and a smaller number of α-smooth muscle actin positive cells, an activation marker of hepatic stellate cells. These cells are one of the key cell types involved in the progression of liver fibrosis, just as they are the principal cellular source of the excess collagen synthesis during hepatic fibrosis. Anti-fibrotic activity also appears to be associated with the antioxidant properties of *M. oleifera* seed extract. This is supported by lower levels of liver malondialdehyde and protein carbonyl, and increased activity of superoxide dismutase, which would reduce the reactive oxygen species that play an important role in the activation of hepatic stellate cells. Interestingly, the effects observed for the ethanolic extract of *M. oleifera* seeds were, in some cases, comparable to those observed for silymarin, a drug known to have hepato-protective and anti-fibrotic properties.

Other studies have reported the ability of *Moringa* seed extract to attenuate the chronic immune-mediated inflammatory responses typical of certain diseases such as asthma [66] and rheumatoid arthritis [67]. Treatment with ethanolic extract of *Moringa* seeds has, indeed, been found to alleviate broncho-alveolar inflammation by decreasing the infiltration of inflammatory cells into the lungs and reducing the secretion of inflammatory mediators into the airways of asthma-induced rats [66]. Similarly, treatment with the ethanolic extract of *M. oleifera* seeds was found to reduce the paw oedema volume, the serum levels of inflammatory mediators and to protect against lymphocytic infiltration, bone destruction and cartilage erosion in the synovial joint, subsequent to the development of arthritis in rats [67].

The oral administration of *M. oleifera* seed powder (750 mg/day, 8 weeks) also reduced nocturnal heart rate and improved cardiac diastolic function in spontaneous hypertensive rats. Moreover, left ventricular anterior wall thickness, interseptal thickness during diastole, and relative wall thickness were reduced after treatment with *M. oleifera* seed powder. Furthermore, a significant reduction in fibrosis in the left ventricle was also observed. However, treatment with *M. oleifera* seed powder did not modify blood pressure [68].

Finally, the extract of *M. oleifera* seeds has also been found to have antidiabetic properties [69]. After inducing type 1 diabetes by injection of streptozotocin (60 mg/kg of body weight), rats simultaneously treated for 4 weeks with *M. oleifera* seed powder had circulating levels of glucose and glycated haemoglobin lower than the diabetic untreated mice. Moreover, after treating diabetic rats with seed powder, histological analysis showed that the pancreatic tissue of diabetic rats was restored to its normal structure, and the histology showed no pathological changes.

## 7. Perspectives and Conclusions

*M. oleifera* is distributed throughout the world across dry tropical areas, and it is a very promising plant from which to produce oil for human consumption and for non-food uses. However, some questions still need to be addressed. One concerns *Moringa* seed yield under high-density orchard design under different growing conditions: this is a priority to promote best farming practices. Another concern is knowledge exchange. Information about available commercial varieties, and their agronomic performance in different environments, is scarce. Thus, the collection and characterization of cultivated varieties, and the setting up of a collaborative network among institutions already working with *M. oleifera* plants would give scientists and producers access to reliable information and materials that could lead to a better development of *Moringa* plantations. In fact, Ojiako et al. [70], in a study conducted in Nigeria, report critical issues in investment, and the production and marketing of *M. oleifera* as an agricultural raw material for industry. These authors claim that the popularization of the plant among farmers does not correspond to a good knowledge of the plant itself, and several issues like land availability, cropping system adjustment, tree management, germplasm management, credit facilities, quality control research and development of seeds for seed production are far beyond the farmers’ “know how”, and still need addressing in order to develop improved *M. oleifera* cultivation. To overcome these hurdles, they emphasize the need to design and implement a sustainable working network: “a sectorial or geographical concentration of enterprises which produces and sells a range of related products and is thus faced with common challenges and opportunities”. Effective management practices and better varieties will improve seed yield and quality, benefiting all the people involved in the production chain.

Given the nutritional composition of the seeds and oil of *M. oleifera*, they could respectively provide a cheap source of protein and a good source of monounsaturated fatty acids of high MUFA/SFA ratio, sterols and tocopherols. However, even from a nutritional point of view, there are still unanswered questions. For example, it is still not known whether the consumption of these products has an effect on the nutritional status, body composition, status of growth and the risk of diseases in populations of developing countries.

Finally, the seeds of *M. oleifera* contain numerous phytochemical compounds with pharmacological activity. However, this activity is reported in the literature only for animal and cellular models, there has been no study of their pharmacological activity in humans. Therefore, it is premature to sustain the use of *M. oleifera* seeds as a natural medicine alternative to traditional therapies. But this knowledge gap should encourage researchers around the world to further their studies on *M. oleifera* so as to obtain clear and definitive information on the human health benefits of consuming the seeds and other plant parts.

In conclusion, the seeds and oil of *M. oleifera* are interesting products for their nutritional composition and their content of bioactive compounds. Their use could have a positive impact on the nutritional status and health of people of developing countries. However, it is essential that further studies be aimed at (i) identifying the best cultivation conditions to maximize plant production and (ii) demonstrating the effects of *Moringa* seed and oil consumption on human health.

## Figures and Tables

**Table 1:** Chemical composition of *Moringa oleifera* seeds (g/100 g of dry weight).

Nutrients	*Moringa oleifera* Seeds		
	Mean	SD	Range
Fat	36.7	2.8	(34.7–40.4)
Proteins	31.4	1.3	(29.4–33.3)
Carbohydrates	18.4	1.4	(16.5–19.8)
Fiber	7.3	0.5	(6.8–8.0)
Ash	6.2	0.9	(4.4–6.9)
Moisture	7.0	1.2	(5.7–8.9)

Values are means ± standard deviations (SD) and ranges of values reported in the literature [3,24,26,27,28,29].

**Table 2:** Physical and chemical characteristics of the oil.

*Moringa oleifera* Oil			
Characteristics	Cold Press ^a^	*n*-Hexane ^b^	Overall
density at 24 °C (mg/mL)	0.901 ± 0.002	0.901 ± 0.011	0.901 ± 0.009
	(0.899–0.904)	(0.881–0.920)	(0.881–0.920)
refractive index (40 °C)	1.462 ± 0.005	1.459 ± 0.004	1.460 ± 0.005
	(1.459–1.470)	(1.455–1.470)	(1.455–1.470)
color red units	1.7 ± 0.5	1.1 ± 0.6	1.3 ± 0.6
	(1.0–2.0)	(ND–2.2)	(ND–2.2)
color yellow units	28.3 ± 2.4	35.8 ± 14.0	33.5 ± 12.0
	(25.0–30.0)	(22.3–70.0)	(22.3–70.0)
smoke point (°C)	203 ± 2	200 ± 1	201 ± 2
	(201–204)	(198–202)	(198–204)
viscosity (mPa × s)	79.5 ± 25.6	52.8 ± 8.1	64.7 ± 21.8
	(43.8–103.0)	(43.6–62.0)	(43.6–103.0)
acidity (% as oleic acid)	1.91 ± 1.13	1.07 ± 1.12	1.33 ± 1.15
	(1.01–3.50)	(0.32–4.00)	(0.32–4.00)
saponification value (mg of KOH/g oil)	189.6 ± 8.0	183.7 ± 4.3	185.5 ± 6.0
	(179.8–199.3)	(178.1–191.2)	(178.1–199.3)
iodine value (g of I/100 g of oil)	66.54 ± 0.97	67.86 ± 1.45	67.46 ± 1.43
	(65.73–67.80)	(65.58–69.45)	(65.58–69.45)

Values are means ± standard deviations and ranges (in parentheses) of values reported in the literature. ^a^ Studies using cold press [21,22,23,31]; ^b^ Studies using solvent extraction [3,21,22,23,26,27,28,29,31]. ND, not detected.

**Table 3:** Fatty acid composition (g/100 g) of oil.

*Moringa oleifera* Oil			
Fatty Acids	Cold Press ^a^	*n*-Hexane ^b^	Overall
Caprylic (C8:0)	0.03 ± 0.01	0.02 ± 0.01	0.03 ± 0.01
	(0.03–0.04)	(ND–0.03)	(ND–0.04)
Myristic (C14:0)	0.15 ± 0.06	0.11 ± 0.07	0.12 ± 0.07
	(0.10–0.24)	(ND–0.24)	(ND–0.24)
Palmitic (C16:0)	5.82 ± 0.39	6.41 ± 0.95	6.25 ± 0.86
	(5.40–6.34)	(5.51–9.07)	(5.40–9.07)
Palmitoleic (C16:1)	1.27 ± 0.15	1.41 ± 0.75	1.37 ± 0.63
	(1.09–1.42)	(0.82–3.44)	(0.82–3.44)
Margaric (C17:0)	0.07 ± 0.03	0.08 ± 0.02	0.07 ± 0.02
	(0.04–0.09)	(0.04–0.09)	(0.04–0.09)
Stearic (C18:0)	4.81 ± 1.09	5.03 ± 1.06	4.97 ± 1.03
	(3.83–5.80)	(2.68–6.00)	(2.68–6.00)
Oleic (C18:1)	73.59 ± 5.00	73.56 ± 2.91	73.57 ± 3.38
	(67.85–79.50)	(67.79–79.50)	(67.79–79.50)
Linoleic (C18:2)	0.55 ± 0.36	0.83 ± 0.41	0.76 ± 0.41
	(ND–0.77)	(ND–1.29)	(ND–1.29)
Linolenic (C18:3)	0.70 ± 1.00	0.36 ± 0.65	0.46 ± 0.74
	(0.20–2.20)	(ND–2.20)	(ND–2.20)
Arachidic (C20:0)	2.99 ± 0.74	3.32 ± 0.69	3.23 ± 0.70
	(2.20–3.72)	(2.14–4.08)	(2.14–4.08)
Gadoleic (C20:1)	1.86 ± 1.25	1.79 ± 0.74	1.81 ± 0.85
	(ND–2.64)	(ND–2.60)	(ND–2.64)
Behenic (C22:0)	5.97 ± 0.69	6.04 ± 0.81	6.02 ± 0.75
	(5.10–6.74)	(4.57–7.10)	(4.57–7.10)
Erucic (C22:1)	0.13 ± 0.02	0.10 ± 0.07	0.11 ± 0.06
	(0.12–0.15)	(ND–0.19)	(ND–0.19)
Lignoceric (C24:0)	ND	0.54 ± 0.77	0.36 ± 0.63
	(ND–ND)	(ND–1.08)	(ND–1.08)
Cerotic (C26:0)	1.02 ± 0.16	0.86 ± 0.49	0.92 ± 0.39
	(0.90–1.21)	(ND–1.18)	(ND–1.21)
% SFA	20.58 ± 2.89	21.40 ± 2.08	21.18 ± 2.24
	(17.24–23.23)	(17.24–23.79)	(17.24–23.79)
% MUFA	76.81 ± 4.11	76.70 ± 2.57	76.73 ± 2.89
	(71.70–80.70)	(71.71–80.70)	(71.70–80.70)
% PUFA	1.25 ± 0.64	1.16 ± 0.48	1.18 ± 0.50
	(0.89–2.20)	(0.41–2.20)	(0.41–2.20)

Values are means ± standard deviations and ranges (in parentheses) of values reported in the literature. ^a^ Studies using cold press [21,22,23,31]; ^b^ Studies using solvent extraction [3,8,21,22,23,26,27,28,29,31,35]. SFA, saturated fatty acids; MUFA, monounsaturated fatty acids; PUFA, polyunsaturated fatty acids.

**Table 4:** Sterol composition of *Moringa oleifera* oil (%).

*Moringa oleifera* Oil			
Sterol Fractions	Cold Press ^a^	*n*-Hexane ^b^	Overall
Cholesterol	0.17 ± 0.03	0.09 ± 0.05	0.12 ± 0.06
	(0.13–0.19)	(ND–0.13)	(ND–0.19)
Brassicasterol	0.06 ± 0.06	0.05 ± 0.03	0.05 ± 0.04
	(ND–0.12)	(ND–0.08)	(ND–0.12)
24-methylenecholesterol	0.61 ± 0.47	0.90 ± 0.43	0.81 ± 0.44
	(0.07–0.91)	(0.08–1.49)	(0.07–1.49)
Campesterol	17.84 ± 5.14	17.26 ± 3.05	17.43 ± 3.48
	(14.03–23.68)	(15.13–23.83)	(14.03–23.83)
Campestanol	0.28 ± 0.25	0.30 ± 0.19	0.29 ± 0.19
	(ND–0.47)	(ND–0.53)	(ND–0.53)
Δ^7^-Campestanol	ND	0.54 ± 0.14	0.54 ± 0.14
	ND	(0.37–0.70)	(0.37–0.70)
Stigmasterol	19.26 ± 3.33	18.59 ± 2.13	18.79 ± 2.37
	(17.27–23.10)	(16.87–23.06)	(16.87–23.10)
Ergostadienol	0.30 ± 0.31	0.30 ± 0.17	0.30 ± 0.21
	(ND–0.61)	(ND–0.39)	(ND–0.61)
Clerosterol	1.23 ± 0.75	1.80 ± 0.68	1.63 ± 0.72
	(0.65–2.08)	(0.62–2.52)	(0.62–2.52)
β-sitosterol	47.17 ± 1.84	47.07 ± 1.99	47.10 ± 1.85
	(45.58–49.19)	(43.65–50.07)	(43.65–50.07)
Stigmastanol	0.89 ± 0.15	0.79 ± 0.16	0.82 ± 0.16
	(0.76–1.05)	(0.53–1.00)	(0.53–1.05)
Δ^5^-avenasterol	8.04 ± 4.97	9.01 ± 2.83	8.72 ± 3.33
	(2.87–12.79)	(2.94–11.61)	(2.87–12.79)
Δ^7^-avenasterol	0.64 ± 0.26	0.79 ± 0.50	0.74 ± 0.43
	(0.45–0.94)	(ND–1.37)	(ND–1.37)
Δ^5,23^-stigmastadienol	1.23	1.23	1.23
	(1.23–1.23)	(1.23–1.23)	(1.23–1.23)
Δ^7,14^-stigmastanol	0.62 ± 0.18	0.52 ± 0.16	0.56 ± 0.16
	(0.52–0.83)	(0.39–0.76)	(0.39–0.83)
28-isoavenasterol	0.52 ± 0.43	0.74 ± 0.43	0.67 ± 0.42
	(0.27–1.01)	(0.25–1.40)	(0.25–1.40)
Δ^7^-stigmastanol	0.58 ± 0.33	0.68 ± 0.24	0.63 ± 0.24
	(0.35–0.81)	(0.51–0.85)	(0.35–0.85)

Values are means ± standard deviations and ranges (in parentheses) of values reported in the literature. ^a^ Studies using cold press [21,22,31]; ^b^ Studies using solvent extraction [3,21,22,26,28,31].

**Table 5:** Tocopherol content (mg/kg) of *Moringa oleifera* oil.

*Moringa oleifera* Oil			
Tocopherols	Cold Press ^a^	*n*-Hexane ^b^	Overall
α-tocopherol	164.2 ± 88.7	121.7 ± 18.4	132.3 ± 41.9
	(101.5–226.9)	(98.4–140.5)	(98.4–226.9)
γ-tocopherol	55.5 ± 22.6	66.7 ± 22.5	63.9 ± 21.5
	(39.5–71.5)	(27.9–93.7)	(27.9–71.5)
σ-tocopherol	146.1 ± 99.6	59.6 ± 7.9	81.2 ± 55.4
	(75.7–216.6)	(48.0–71.2)	(48.0–216.6)

Values are means ± standard deviations and ranges (in parentheses) of values reported in the literature. ^a^ Studies using cold press [21,31]; ^b^ Studies using solvent extraction [21,26,27,28,29,31].

**Table 6:** Bioactive compounds in *M. oleifera* seeds.

Compounds	Reference	Compounds	Reference
**Alkaloids**		**Glycosides**	
Moringine	[48]	Strophantidin	[49]
		4-(α-l-rhamnosyloxy)benzyl isothiocyanate	[46,50]
**Flavonoids**		4-(4’-*O*-acetyl-α-l-rhamnosyloxy)benzyl isothioyanate	[51]
Catechin	[47]	4-(β-d-glucopyranosyl-1→4-α-l-rhamnopyranosyloxy)benzyl thiocarboxamide	[52,53]
Epicatechin	[47]	4-*O*-(α-l-rhamnosyloxy)benzyl glucosinolate	[54]
Quercetin	[47]	4-(α-l-rhamnopyranosyloxy)-benzylglucosinolate	[55]
Kaempferol	[45]	Niazimicin	[46,51]
		4-(α-l-rhamnosyloxy)benzyl acetonitrile (niazirin)	[46]
**Phenolic acids**		*O*-ethyl-4-(α-l-rhamnosyloxy)benzyl carmate	[46]
Gallic acid	[45,47]	Glycerol-1-1-(9-octadecanoate)	[46]
*p*-Coumaric acid	[47]	3-*O*-(6’-*O*-oleoyl-β-d-glucopyranosyl)-β-sitosterol	[46]
Ferulic acid	[47]	β-sitosterol-3-*O*-β-d-glucopyranoside	[46]
Caffeic acid	[47]	3-Hydroxy-4-(α-l-rhamnopyranosyloxy)benzyl glucosinolate	[56]
Protocatechuic acid	[47]	4-(2/3/4′-*O*-acetyl-α-l-rhamnopyranosyloxy)benzyl glucosinolate	[56]
Cinnamic acid	[47]	Glucosinalbin	[56]
Ellagic acid	[45]	Glucoraphanin	[56]
		Glucoiberin	[56]

## Abbreviations

MUFA/SFA	Monounsaturated/Saturated fatty acids

## References

[1] Leone A., Spada A., Battezzati A., Schiraldi A., Aristil J., Bertoli S. (2015). Cultivation, genetic, ethnopharmacology, phytochemistry and pharmacology of *Moringa oleifera* leaves: An overview. Int. J. Mol. Sci..

[2] Leone A., Fiorillo G., Criscuoli F., Ravasenghi S., Santagostini L., Fico G., Spadafranca A., Battezzati A., Schiraldi A., Pozzi F. (2015). Nutritional characterization and phenolic profiling of *Moringa oleifera* leaves grown in chad, sahrawi refugee camps, and haiti. Int. J. Mol. Sci..

[3] Anwar F., Ashraf M., Bhanger M.I. (2005). Interprovenance variation in the composition of *Moringa oleifera* oilseeds from pakistan. J. Am. Oil Chem. Soc..

[4] Ndabigengesere A., Subba Narasiah K. (1998). Quality of water treated by coagulation using *Moringa oleifera* seeds. Water Res..

[5] Emmanuel S.A., Emmanuel B.S., Zaku S.G., Thomas S.A. (2011). Biodiversity and agricultural productivity enhancement in nigeria: Application of processed *Moringa oleifera* seeds for improved organic farming. Biol. J. N. Am..

[6] Biodiesel C., Centre for Jatropha Promotion Enhanced Moringa Agronomy: *Moriniga* Research & Revelopment. http://www.jatrophabiodiesel.org/moringa/agronomy.php.

[7] Sánchez N., Ledin S., Ledin I. (2006). Biomass production and chemical composition of *Moringa oleifera* under different management regimes in nicaragua. Agrofor. Syst..

[8] Ayerza R. (2011). Seed yield components, oil content, and fatty acid composition of two cultivars of moringa (*Moringa oleifera* Lam.) growing in the Arid Chaco of Argentina. Ind. Crops Prod..

[9] Muhl Q.E., du Toit E.S., Steyn J.M., Apostolides Z. (2014). Bud development, flowering and fruit set of *Moringa oleifera* Lam. (horseradish tree) as affected by various irrigation levels. J. Agric. Rural Dev. Trop. Subtrop..

[10] Dania S.O., Akpansubi P., Eghagara O.O. (2014). Comparative effects of different fertilizer sources on the growth and nutrient content of *Moringa* (*Moringa oleifera*) seedling in a greenhouse trial. Adv. Agric..

[11] Isaiah M.A. (2013). Effects of inorganic fertilizer on the growth and nutrient composition of *Moringa* (*Moringa oleifera*). J. Emerg. Trends Eng. Appl. Sci..

[12] Heuzé V., Tran G., Hassoun P., Bastianelli D., Lebas F. Moringa (Moringa oleifera).

[13] Bhattacharya A., Mandal S. (2004). Pollination, pollen germination and stigma receptivity in *Moringa oleifera* lamk. Grana.

[14] Foidl N., Makkar H.P.S., Becker K., Fuglie L.J. (2001). The potential of *Moringa oleifera* for agricultural and industrial uses. The Miracle Tree: The Multiple Attributes of Moringa.

[15] Rajangam J., Azahakia Manavalan R.S., Thangaraj T., Vijayakumar A., Muthukrishan N. Status of Production and Utilisation of *Moringa* in Southern India. Proceedings of the International Conference on Development Potential for *Moringa* Products.

[16] Ndubuaku U.M., Ndubuaku T.C.N., Ndubuaku N.E. (2014). Yield characteristics of *Moringa oleifera* across different ecologies in nigeria as an index of its adaptation to climate change. Sustain. Agric. Res..

[17] Kumar A.R., Prabhu M., Ponnuswami V., Lakshmanan V., Nithyadevi A. (2014). Scientific seed production techniques in *Moringa*. Agric. Rev..

[18] Ayerza R. (2012). Seed and oil yields of *Moringa oleifera* variety periyakalum-1 introduced for oil production in four ecosystems of south america. Ind. Crops Prod..

[19] Suthanthirapandian I.R., Sambandamurthy S., Irulappan I. (1989). Variations in seedling populations of annual moringa (*Moringa pterygosperma* gaertn.). South Indian Hortic..

[20] Ramachandran C., Peter K.V., Gopalakrishnan P.K. (1980). Drumstick (*Moringa oleifera*): A multipurpose Indian vegetable. Econ. Bot..

[21] Tsaknis J., Lalas S., Gergis V., Spiliotis V. (1998). A total characterisation of *Moringa oleifera* malawi seed oil. Riv. Ital. Delle Sostanze Grasse.

[22] Lalas S., Tsaknis J. (2002). Characterization of *Moringa oleifera* seed oil variety “periyakulam 1”. J. Food Compos. Anal..

[23] Ogunsina B.S., Indira T.N., Bhatnagar A.S., Radha C., Debnath S., Gopala Krishna A.G. (2014). Quality characteristics and stability of *Moringa oleifera* seed oil of Indian origin. J. Food Sci. Technol..

[24] Oliveira J.T.A., Silveira S.B., Vasconcelos I.M., Cavada B.S., Moreira R.A. (1999). Compositional and nutritional attributes of seeds from the multiple purpose tree *Moringa oleifera* lamarck. J. Sci. Food Agric..

[25] Santos A.F., Argolo A.C., Coelho L.C., Paiva P.M. (2005). Detection of water soluble lectin and antioxidant component from *Moringa oleifera* seeds. Water Res..

[26] Anwar F., Bhanger M.I. (2003). Analytical characterization of *Moringa oleifera* seed oil grown in temperate regions of pakistan. J. Agric. Food Chem..

[27] Anwar F., Nahid Zafar S., Rashid U. (2006). Characterization of *Moringa oleifera* seed oil from drought and irrigated regions of Punjab, Pakistan. Grasas Y Aceites.

[28] Anwar F., Rashid U. (2007). Physico-chemical characteristics of *Moringa oleifera* seeds and seed oil from a wild provenance of Pakistan. Pak. J. Bot..

[29] Rahman I.M.M., Barua S., Nazimuddin M., Begum Z.A., Rahman M.A., Hasegawa H. (2009). Physicochemical properties of *Moringa oleifera* Lam. Seed oil of the indigenous-cultivar of bangladesh. J. Food Lipids.

[30] Boskou D. (2011). Olive oil. Vegetable oils in Food Technology.

[31] Tsaknis J., Lalas S., Gergis V., Dourtoglou V., Spiliotis V. (1999). Characterization of *Moringa oleifera* variety mbololo seed oil of Kenya. J. Agric. Food Chem..

[32] Sengupta A., Gupta M.P. (1970). Studies on the seed fat composition of moringaceae family. Fette Seifen Anstrichm..

[33] Schwingshackl L., Hoffmann G. (2014). Monounsaturated fatty acids, olive oil and health status: A systematic review and meta-analysis of cohort studies. Lipids Health Dis..

[34] Gnagnarella P., Salvini S., Parpinel M. Food Composition Database for Epidemiological Studies in Italy. http://www.bda-ieo.it.

[35] Rashid U., Anwar F., Moser B.R., Knothe G. (2008). *Moringa oleifera* oil: A possible source of biodiesel. Bioresour. Technol..

[36] Abumweis S.S., Barake R., Jones P.J. (2008). Plant sterols/stanols as cholesterol lowering agents: A meta-analysis of randomized controlled trials. Food Nutr. Res..

[37] Demonty I., Ras R.T., van der Knaap H.C., Duchateau G.S., Meijer L., Zock P.L., Geleijnse J.M., Trautwein E.A. (2009). Continuous dose-response relationship of the LDL-cholesterol-lowering effect of phytosterol intake. J. Nutr..

[38] Ras R.T., Geleijnse J.M., Trautwein E.A. (2014). LDL-cholesterol-lowering effect of plant sterols and stanols across different dose ranges: A meta-analysis of randomised controlled studies. Br. J. Nutr..

[39] Gupta R., Sharma A.K., Dobhal M.P., Sharma M.C., Gupta R.S. (2011). Antidiabetic and antioxidant potential of β-sitosterol in streptozotocin-induced experimental hyperglycemia. J. Diabetes.

[40] Genser B., Silbernagel G., de Backer G., Bruckert E., Carmena R., Chapman M.J., Deanfield J., Descamps O.S., Rietzschel E.R., Dias K.C. (2012). Plant sterols and cardiovascular disease: A systematic review and meta-analysis. Eur. Heart J..

[41] Manzoor M., Anwar F., Iqbal T., Bhanger M.I. (2007). Physico-chemical characterization of *Moringa* concanensis seeds and seed oil. J. Am. Oil Chem. Soc..

[42] Tsaknis J. (1998). Characterisation of *Moringa peregrina* Arabia seed oil. Grasas Y Aceites.

[43] Biswas W.K., John M.B. (2008). Life Cycle Assessment of Biodiesel Production from Moringa Oleifera Oilseeds.

[44] Karmakar A., Karmakar S., Mukherjee S. (2010). Properties of various plants and animals feedstocks for biodiesel production. Bioresour. Technol..

[45] Singh B.N., Singh B.R., Singh R.L., Prakash D., Dhakarey R., Upadhyay G., Singh H.B. (2009). Oxidative DNA damage protective activity, antioxidant and anti-quorum sensing potentials of *Moringa oleifera*. Food Chem. Toxicol..

[46] Guevara A.P., Vargas C., Sakurai H., Fujiwara Y., Hashimoto K., Maoka T., Kozuka M., Ito Y., Tokuda H., Nishino H. (1999). An antitumor promoter from *Moringa oleifera* Lam.. Mutat. Res./Genet. Toxicol. Environ. Mutagen..

[47] Govardhan Singh R.S., Negi P.S., Radha C. (2013). Phenolic composition, antioxidant and antimicrobial activities of free and bound phenolic extracts of *Moringa oleifera* seed flour. J. Funct. Foods.

[48] Kirtikar K.R., Basu B.D. (1975). Indian Medicinal Plants.

[49] Olayemi A.B., Alabi R.O. (1994). Studies on traditional water purification using *Moringa oleifera* seeds. Afr. Stud. Monogr..

[50] Eilert U., Wolters B., Nahrstedt A. (1981). The antibiotic principle of seeds of *Moringa oleifera* and moringa stenopetala. Planta Med..

[51] Faizi S., Siddiqui B.S., Saleem R., Siddiqui S., Aftab K., Gilani A.H. (1994). Isolation and structure elucidation of new nitrile and mustard oil glycosides from *Moringa oleifera* and their effect on blood pressure. J. Nat. Prod..

[52] Oluduro O.A., Aderiye B.I., Connolly J.D., Akintayo E.T., Famurewa O. (2010). Characterization and antimicrobial activity of 4-(β-d-glucopyranosyl-1→4-α-l-rhamnopyranosyloxy)-benzyl thiocarboxamide; a novel bioactive compound from *Moringa oleifera* seed extract. Folia Microbiol. (Praha).

[53] Maiyo F.C., Moodley R., Singh M. (2016). Cytotoxicity, antioxidant and apoptosis studies of quercetin-3-*O* glucoside and 4-(β-d-glucopyranosyl-1→4-α-l-rhamnopyranosyloxy)-benzyl isothiocyanate from *Moringa oleifera*. Anticancer Agents Med. Chem..

[54] Amaglo N.K., Bennett R.N., Curto R.B.L., Rosa E.A.S., Turco V.L., Giuffrida A., Curto A.L., Crea F., Timpo G.M. (2010). Profiling selected phytochemicals and nutrients in different tissues of the multipurpose tree *Moringa oleifera* L., grown in Ghana. Food Chem..

[55] Bennett R.N., Mellon F.A., Foidl N., Pratt J.H., Dupont M.S., Perkins L., Kroon P.A. (2003). Profiling glucosinolates and phenolics in vegetative and reproductive tissues of the multi-purpose trees *Moringa oleifera* L. (horseradish tree) and *Moringa stenopetala* L.. J. Agric. Food Chem..

[56] Maldini M., Maksoud S.A., Natella F., Montoro P., Petretto G.L., Foddai M., de Nicola G.R., Chessa M., Pintore G. (2014). *Moringa oleifera*: Study of phenolics and glucosinolates by mass spectrometry. J. Mass Spectrom..

[57] Popoola J.O., Obembe O.O. (2013). Local knowledge, use pattern and geographical distribution of *Moringa oleifera* Lam. (Moringaceae) in nigeria. J. Ethnopharmacol..

[58] Padla E.P., Solis L.T., Levida R.M., Shen C.C., Ragasa C.Y. (2012). Antimicrobial isothiocyanates from the seeds of *Moringa oleifera* Lam.. Z. Naturforsch. C.

[59] Jeon S.R., Lee K.H., Shin D.H., Kwon S.S., Hwang J.S. (2014). Synergistic antimicrobial efficacy of mesoporous zno loaded with 4-(α-l-rhamnosyloxy)-benzyl isothiocyanate isolated from the *Moringa oleifera* seed. J. Gen. Appl. Microbiol..

[60] Lurling M., Beekman W. (2010). Anti-cyanobacterial activity of *Moringa oleifera* seeds. J. Appl. Phycol..

[61] Galuppo M., Nicola G.R., Iori R., Dell’utri P., Bramanti P., Mazzon E. (2013). Antibacterial activity of glucomoringin bioactivated with myrosinase against two important pathogens affecting the health of long-term patients in hospitals. Molecules.

[62] Williams L.L. (2013). *Moringa olefiera*: Could this be an answer to our need for an alternative to fighting drug-resistance and chronic infections?. Med. Aromat. Plants.

[63] Al-Asmari A.K., Albalawi S.M., Athar M.T., Khan A.Q., Al-Shahrani H., Islam M. (2015). *Moringa oleifera* as an anti-cancer agent against breast and colorectal cancer cell lines. PLoS ONE.

[64] Elsayed E.A., Sharaf-Eldin M.A., Wadaan M. (2015). In vitro evaluation of cytotoxic activities of essential oil from *Moringa oleifera* seeds on HeLa, HepG2, MCF-7, CACO-2 and L929 cell lines. Asian Pac. J. Cancer Prev..

[65] Hamza A.A. (2010). Ameliorative effects of *Moringa oleifera* Lam seed extract on liver fibrosis in rats. Food Chem. Toxicol..

[66] Mahajan S.G., Mali R.G., Mehta A.A. (2007). Effect of *Moringa oleifera* Lam. Seed extract on toluene diisocyanate-induced immune-mediated infla mmatory responses in rats. J. Immunotoxicol..

[67] Mahajan S.G., Mali R.G., Mehta A.A. (2007). Protective effect of ethanolic extract of seeds of *Moringa oleifera* Lam. Against infla mmation associated with development of arthritis in rats. J. Immunotoxicol..

[68] Randriamboavonjy J.I., Loirand G., Vaillant N., Lauzier B., Derbre S., Michalet S., Pacaud P., Tesse A. (2016). Cardiac protective effects of *Moringa oleifera* seeds in spontaneous hypertensive rats. Am. J. Hypertens..

[69] Al-Malki A.L., El Rabey H.A. (2015). The antidiabetic effect of low doses of *Moringa oleifera* Lam. Seeds on streptozotocin induced diabetes and diabetic nephropathy in male rats. BioMed Res. Int..

[70] Ojiako F.O., Adikuru N.C., Emenyonu C.A. (2011). Critical issues in investment, production and marketing of moringa oleifera as an industrial agricultural raw material in nigeria. J. Agric. Res. Dev..

